# Perceptions of professional nurses regarding introduction of the Batho Pele principles in State hospitals

**DOI:** 10.4102/curationis.v38i1.1128

**Published:** 2015-03-09

**Authors:** Sindiwe James, Thenjiwe M. Miza

**Affiliations:** 1 Department of Nursing Science, North Campus, Nelson Mandela Metropolitan University, Port Elizabeth, South Africa; 2 Livingstone Hospital, Port Elizabeth, South Africa

## Abstract

**Background:**

The South African health care delivery system has shifted focus to primary health care since 1994. For this purpose the Batho Pele principles were introduced. Nurses claim, however, that since the introduction of these principles patients and their families have been making unnecessary and sometimes impossible demands of nursing staff. This article presents the perceptions of the professional nurses regarding the introduction of the Batho Pele principles in their workplace.

**Objectives:**

To describe the perceptions of professional nurses regarding introduction of the Batho Pele principles and to recommend guidelines to facilitate measures to realise the objects of these principles.

**Method:**

A qualitative, exploratory, descriptive and contexual research design was used. Six audio-taped focus group discussions and field notes were used to collect data from purposively sampled participants who have worked in the outpatient departments of hospitals in the Port Elizabeth Hospital Complex. Guba's model of trustworthiness was used to confirm integrity of the study, whilst the participants were kept anonymous, protected from harm and participated voluntarily. Data analysis was done using Tesch's data analysis spiral and with the involvement of an independent-coder.

**Results:**

Three themes emerged, revealing that the professional nurses perceived the objectives of the Batho Pele principles as difficult to uphold due to the inadequate planning prior to their implementation. Inadequacy of human and material resources aggravated this perception.

**Conclusion:**

Professional nurses are not happy with how things are in terms of introduction of the Batho Pele principles, but are optimistic of a positive change in the near future.

## Introduction and background

The quality of care rendered to patients has always been the main focus of health care, and yet this still remains a challenge to State hospitals in South Africa (SA). Quality of care refers to the attributes or characteristics of excellence, with an emphasis on the principles of applicability and acceptability (Muller 2009). If these principles are adhered to, quality healthcare delivery can be achieved. Patients associate ‘quality of care’ with the quality of care that they receive as well as the amount of empathy shown towards them by nursing staff (Booyens 2008).

In recent years the rendering of quality care in South African State hospitals has been compounded by the resignations of senior and experienced nurses in all sectors, including outpatient departments of different hospital units. This brain and skill ‘drain’ happened at the same time as inception of primary healthcareopportunities for all in SA. Primary health care was seen as the key element in the Government's plan to transform the health services in SA and to bridge healthcare service delivery gaps where possible (Muller 2009). In order to accomplish this a White Paper on the Batho Pele principles was adopted and published in 1997 as a framework for provision of service delivery by the Department of Health (DoH) (DoH 2001) The core value of these principles, as stated in the English translation, is that people are made a priority. The aim is to ensure that healthcare providers are sensitive to patients’ healthcare needs. These principles, which guide healthcare providers to observe standards of consultation, service standards, access, courtesy, information, openness and transparency, redress and value for money (Muller 2009) are discussed next.

### The Batho Pele principles

These prinicples are almost synonymous and therefore difficult to view separately. For the purposes of optimal discussion they will therefore be grouped accordingly.

#### Standards of consultation, service, access and courtesy

The above principles insist that citizens should be *consulted* regarding the level and quality of the public service they receive. Should the need arise, they should be further referred to gain *access* to the correct healthcare facility. Here the emphasis is that patients know how healthcare services are classified to ensure that they receive prompt care. As those who have easy access to this information, healthcare workers (for the purposes of this article, professional nurses) should frequently inform the patients in this regard, be tolerant to their inquiries and be respectful of patients as individuals. Such actions will enhance the realisation of effective access to healthcare services.

The principle of access in terms of the Batho Pele principles means that patients should have equal opportunities in terms of resources and facilities (DoH1997, in Muller 2009). In this regard the DoH has vowed to promote and improve access to healthcare facilities through provision of comprehensive healthcare services within a 5 km radius of catchment areas at all times (DoH 2001). However, access may go beyond the designated catchment areas, and hence the need at times for a further referral.

#### Information, openness and transparency, redress and value for money

Besides consultation, access and courtesy, the Batho Pele principles also make reference to the provision of fully accurate information to citizens about the public health services to which they are entitled. The public have faith that the professional nurse will give them accurate information regarding health issues; therefore the nurse has the responsibility to ensure that (s)he is aware of all changes and new developments in the healthcare sector. Whilst sharing information with patients, healthcare workers (professional nurses) should observe the culture of openness and transparency. Furthermore, the Batho Pele principles determine that public services should be provided economically and efficiently in order to provide citizens with the best possible service for the value of their money.

Having mentioned the various principles that comprise the notion of Batho Pele, one must also remember that these are informed by three sets of beliefs: care, teamwork and service.

### Care, teamwork and service

Caring for patients in outpatient departments cannot be done in isolation of other healthcare providers and relevant stakeholders. Teamwork and support is of even more importance in an outpatient department for the sake of interdependence and good-quality service. Covey (1989) states that interdependence opens up worlds of possibilities for deep, rich, meaningful associations and increased productivity. As mentioned earlier, some patients will access the healthcare service as referrals from other service centres or will need to be referred to other services. The procedure relating to referral of patients requires consultation of expert knowledge as well as coordination of all processes involved, hence the need for efficient teamwork.

The concept of teamwork becomes more appreciated in health care as it brings with it the experience of interdependence, complementary skills, information sharing and making decisions together towards the set goals of the unit (Muller, Bezuidenhout & Jooste 2011:334). In most hospitals a multidisciplinary team is formed whereby all the health care disciplines meet to discuss the care and referral needs of their patients. Booyens (2008) states that the aim of this teamwork is to determine priorities and clarify roles and responsibilities which, in the context of this article, are significant in promoting quality healthcare service.

In some instances, as is the case in the hospital where the co-author of this article works, the same outpatient department's patients are referred from clinic to clinic to satisfy their healthcare needs. This action may be confusing and annoying to some patients. Explaining the process of referral in a caring manner to patients is therefore a crucial role of professional nurses in identifying patient concerns, problems related to referral systems, possible conflict situations and improvement of communication between team members and community.

Nurses as messengers of either bad or good healthcare-related information are sometimes challenged with being non-caring, especially by patients who do not want to hear the bad news or results related to their health (Huber 2014:120). Patients usually become confused when they do not understand medical instructions, and thus come into conflict with the healthcare person (Huber 2014:120), as in the case of the referral system brought about by introduction of the Batho Pele principles. Doctors and professional nurses should be mindful of the consequences of miscommunication and always try to be as clear in their instructions as possible. Muller (2009) asserts that caring is integral to the ethical and philosophical foundation of nursing. Nurses join the nursing profession with a vision to serve society, this vision being driven by the values of passion and caring which could be enhanced through practising the Batho Pele principles.

Searle (2006) states that when nurses are admitted to the professional register they declare publicly that they are competent and willing to meet the standards of nursing required from them, and that they will dedicate themselves to serving people through meeting their nursing needs. The eight Batho Pele principles and belief sets can easily be applied within patient nursing care by professional nurses working in outpatient departments, as these are also entrenched in the message of caring as translated through the Pledge of Service. Fundamental to adequately serving the patient are the principles of responsibility and accountability, as well as a willingness to go beyond the call of duty, for which the Batho Pele principles chiefly stand. Professional nurses are held responsible for their actions in their day-to-day activities, and despite the conditions under which they serve, their duties include being held accountable for caring for their patients.

### Problem statement

The researcher is exposed to nurses who almost daily express concern that, since introduction of the Batho Pele principles, patients have become abusive towards them, making their work unbearable. According to these nurses, patients seem to have unrealistic demands due to their expectations having been raised by these principles. Some of these nurses feel that it is not easy to uphold the principle of access, for example when there is a shortage of staff, especially skilled staff, and a shortage of medications.

On average, one professional nurse takes care of 20 patients a day in an outpatients department. These patients, as reported by the professional nurses working in the outpatient departments, present themselves to the department as early as 06h00 and might leave between 14h00 and 16h00. This delay is caused mainly by limited material and human resources.

Lack of material resources may at times include a lack of medicines to be given to patients, as well as a lack of diagnostic equipment. Nurses are confronted with the anger of patients who are exposed to such supply shortages. As the nursing category that carries the bulk of patient care in the outpatient departments, Professional nurses bear the brunt of the bad behaviour of patients and feel that the authorities do not listen when they report abusive tendencies or behaviour of patients.

Furthermore, there have been many negative reports in the media in recent years regarding the ‘bad attitude’ of nurses towards patients and their relatives, as well as about the substandard housekeeping and nursing care in State hospitals. Professional nurses expressed concern about these reports.

This study was designed to answer the following research question: What is the perception of professional nurses regarding the introduction of the Batho Pele principles?

#### Research objectives

The objectives of the study were to: describe the perceptions of professional nurses regarding the introduction of the Batho Pele principles; and to recommend guidelines to facilitate measures for all parties involved in realisation of the objects of the Batho Pele principles.

## Research design

The research design for the study was qualitative, exploratory, descriptive and contextual in nature. The design suited the study as it allowed the researcher to engage with and to fully understand the lived experiences of the participants (Johnston & Onwuegbuzie 2004:20).

The population for the study was professional nurses who had been working in the outpatient departments of the three hospitals in the Port Elizabeth Hospital Complex within the Nelson Mandela Metropolitan Municipality for at least five years. Specific inclusion criteria were at least five years’ experience of working in the outpatients department in a State hospital; work in either the adult or children outpatients department; and knowledge and practical skills which meant that they should possess a better understanding of what nursing stands for, and enhanced the purposive sampling of participants for this study (Parahoo 2006). Forty professional nurses were sampled; only 32 participated as the remaining 8 were either sick or not on duty on the day of data collection.

### Data collection methods

Data were collected through six audio-taped focus group discussions (FGDs) with no less than four participants each, as well as the taking of field notes. Of these six FGDs, two were for the purposes of a pilot study. No errors were identified in the data from the pilot study, and therefore data collected from these two groups were also included in the data analysis and results. Following the discussion with regard to the pilot study, with no major concerns identified, the researcher continued with data collection (Hornby 2005).

The FGDs were scheduled to last no longer than 45 uninterrupted minutes, but at times this schedule was not easy to maintain due to the nature of engagement with the participants, and they would be extended by 5–10 minutes. The interviews were based on one central question, with subsequent questions developing from the responses of the group members, and a set of two predetermined questions.

The central question was: Tell me, how do you feel about the introduction of Batho Pele principles in your institution? The predetermined questions were: What would you tell me about the implementation of the Batho Pele principles?; and What do you think could assist with the implementation of the Batho Pele principles?

The FGDs were conducted over a period of seven days during March 2010. As the researcher had no previous experience in conducting research interviews, the supervisor of the study assisted with facilitation of the FGD with one group for the pilot study and two of the main study FGDs. This facilitation was mainly to teach the researcher how to prepare participants before commencing the interview and to direct a question to a specific participant using the name code before or after the question has been asked, so as to allow individual responses. The researcher was also guided in keeping the focus to the research questions. Data saturation was the indication to discontinue data collection and complete the data analysis process (Burns & Grove 2011; Green & Thorogood 2009).

## Ethical considerations

The researchers were mindful of meeting all ethical considerations (Silverman 2011). A complete research proposal was presented to the research committee of the Nursing Science Department for approval. Written permission to conduct research was obtained from the Research, Technology and Innovation Committee of the Faculty of Health Sciences, with ethical clearance from the Ethical Committee of Nelson Mandela Metropolitan University. Furthermore, the DoH via the Chief Executive Officer of the Port Elizabeth Hospital Complex gave permission to use the hospitals as the site for conducting the study. Informed consent was obtained from the individual participants, as well as permission to identify them using codes such as P1 or P5.

Other ethical issues that were adhered to included preventing harm, no deception of participants, confidentiality and anonymity (De Vos *et al*. 2013). Participants were further assured of anonymity and confidentiality of their information and their privacy. Name codes were used to identify the tapes and transcripts and none of the participants’ particulars were divulged. In order not to cause harm, each FGD was conducted in a sensitive manner (Babbie 2010). Participants maintained their right to withdraw at any stage of the study without any penalties.

### Trustworthiness

Babbie and Mouton (1998) state that the eminence of any qualitative study is determined by the level of trustworthiness it ‘portrays’. To ensure valid results and trustworthiness, the researcher used Guba's model as described by Krefting (1991). The focus of the model is truth value, applicability, consistency and neutrality. Credibility was assured through member checking and the use of peer examination reflexivity, whilst confirmability was maintained through an audit trail.

Transferability refers to the ability to apply the findings of the study in another context or to other participants. The activities used to enhance transferability include a thick description, achieved by collecting and providing sufficient detailed data within the context of the study, purposive sampling, and data saturation. Dependability was maintained by use of a code-recode procedure and peer examination.

## Results and discussion

Participants were between the ages of 35 and 50years. The participants were all black people with most being Xhosa-speaking, whilst a few were mixed-race. Two groups had one male participant and two groups had one mixed-race participant. The male participants represented 100% of the male professional nurses in the department chosen as the research site.

There were no white employees at these research sites, and hence no white participants. In SA most townships – the environment in which the study was conducted – are predominantly populated by Xhosa families and individuals, whilst the nursing profession is a predominantly female-dominated profession.

Data were analysed using Tesch's method as stated in Creswell (2008:154) and by making use of an independent-coder. Three main themes and several sub-themes and categories emerged from the data analysis.

### Theme 1: Lack of planning with the introduction of Batho Pele

Participants indicated positive and negative emotions regarding introduction of the Batho Pele principles. The participants felt strongly that introduction of the Batho Pele principles lacked a sound foundation to their implementation. Said one participant: ‘My experience with Batho Pele principles is that it was designed to transform the health services, the aim was good, but the government fails to do the ground work.’

As a result, the Batho Pele principles could not meet the set positive objectives of providing quality service delivery or of developing competent, committed and professional health workers for whom people or patients come first (DoH 2008).

According to the participants a lack of planning was the chief shortcoming with introduction of the Batho Pele principles: ‘No planning was done before implementing the Batho Pele principles’. The participants felt that proper planning to implement the principles would have assisted the authorities to anticipate challenges and devise strategies to deal with such challenges. The experience of limited planning before implementing the principles was expressed as follows: ‘… without checking on the consequences the patients were informed of the Batho Pele principles. Now the patients want to hear what they want to hear’. Adequate planning would also have afforded well thought-through processes to correctly inform the public of the introduction of the Batho Pele principles.

The Batho Pele principles were meant to bring about a change in the usual manner of healthcare service delivery in the country. The environmental dynamics related to the change process, namely emotional involvement and resistance, were therefore to be observed (Jooste 2009; Huber 2010). Planning allows involvement of all stakeholders, which according to this study are the professional nurses and the community, thus encouraging ownership (Jooste 2009). Planned change enhances productivity and lessens the amount of individual stress resulting from the stakeholders’ efforts to cope (Huber 2010).

According to the participants, the patients and the public are largely misinformed about the Batho Pele principles. As one put it: ‘Some of these patients are not educated. These principles were read to them then they picked up what they want to hear and interpret it wrongly.’

As a result, they are inclined to make inappropriate demands, such as self-referring to an institution of a higher level of care instead of being referred from a nearby clinic or private doctor. Healthcare services were not rendered previously in terms of different levels; this resulted in duplication of services being the order of the day. Hospitals were providing primary to secondary services simultaneously – thus patients could present for a consultation and then would either be admitted and managed further or referred from one service to another. The professional nurses felt that the newly introduced referral system is not easy for patients and their families to understand.

Availability of medicines and prescriptions are currently categorised according to the level of care of that particular institution, and at times are out of stock. These are demanded by the patients from the professional nurses, although it is a managerial responsibility to order medicines. When the participants explained the impossibility of certain patient demands and informed patients of the correct procedure to be followed, the patients and their relatives became angry – shouting and accusing them of being uncaring and selfish. In this regard one of the participants expressed herself as follows:

‘Patients shout and threaten us. You become scared and humiliated because patients shout at you in front of colleagues and other patients.’

Some even threatened to channel their dissatisfaction through the media, which would cause even worse conflict between the professional nurses and their patients.

Informing and explaining the merits of change to subordinates and followers enhances the process of organisational change (Jooste 2009). Here the ‘followers’ – the community members – seemed to misunderstand the practical meaning of the ‘right to access of health care’ due to a lack of adequate explanation regarding the full scope and meaning of this ‘right’. Below are examples of expressions bythe participants with regard to limited understanding of the ‘right to access of health care’ by the community:

‘Patients are not aware that other things are beyond the control of a nurse.’

‘Patients are rude. They have taken this the wrong way. They think when they shout at a nurse it is their right. They think that these rights are to disrespect and abuse nurses.’

Further mixed emotions were experienced by study participants because they felt that implementation of the policy was further grossly affected by a shortage of human and material resources:

‘Nurses would like to work and produce good results. When there are no resources I feel disgruntled, fed up. I feel like taking my bag and resign[*ing*] and find[*ing*] work where there are resources and I can give optimal care.’

‘Another thing that makes us bad to the public is shortage of resources …’

‘You feel embarrassed when you must re-book a patient because of no availability of resources. Some of these patients are poor. At times for most of these patients they have kept that money to come to the hospital; some have borrowed the money, and they must go back without any help because there are no resources.’

Both proper planning and adequate availability of resources are needed to influence achievement of the principles’ goals. Despite existing disparities between policy and implementation, healthcare professionals cannot refuse to practice, because they are bound by professional ethics and the Pledge of Service that charges South African nurses to protect the dignity of their patients and families and the community at large, and to protect and maintain a safe and therapeutic health care environment, regardless of colour and creed, based on the human needs of the patient.

#### Sub-theme 1.1: Difficulty in upholding the policy of the Batho Pele principles

Participants expressed concern that because of a lack of planning in implementing and incorporating the Batho Pele principles into policy, it is sometimes difficult for them to act in an ethical manner in response to the way in which patients and community members address or treat them:

‘I can say Batho Pele principles is something good introduced by the government but sometimes it is difficult to uphold.’

‘It is a good tool, but patients use it the wrong way.’

Ethics are vital in nursing practice and any occupation dealing with human life needs, both to regulate the practitioner so as to protect the good name of the profession, and to protect the public (Huber 2010). As aforementioned, at the core of provision of nursing is the value of and respect for human life. Professional nurses found it difficult to uphold this value as introduction of the principles did not fully accommodate issues related to human and material supplies, resulting in negative responses from the patients and community.

Burns and Grove (2009) define ethics as a means of endeavouring towards realistic outcomes where others are involved. Ethics determine that which is ‘good’ and ‘bad’, ‘right’ or ‘wrong’, or a moral duty and obligation (Pope & May 2005).

In this regard Burns and Grove (2009) maintain that problems of ethics relate to conscience, justice and responsibility, amongst others. The BathoPele principles were formulated to ensure that patients are treated with respect and consideration, and that the healthcare providers show courtesy and respect when interacting with patients. With this understanding in mind, professional nurses often have to keep quiet and ignore abusive behaviour from their patients, despite feeling heartbroken.

#### Sub-theme 1.2: Batho Pele principles not planned properly

Change is complicated and should therefore be planned and implemented properly. If proper planning is not done, it leads to frustration, confusion and resistance amongst employees (Tomey 2009). Planning, according to Booyens (2008), is the cornerstone of good management and is grounded in the vision, mission, goals, philosophy and objectives of the organisation. Therefore, when planning for change the manager should not settle on a change approach before first examining the problem, the personal environment, time constraints, resources and goals to be achieved (Hellriegel *et al*. 2002). In the opinion of the participants this approach should have been adopted by the health authorities before their implementation of the Batho Pele principles:

‘No planning was done before implementing the Batho Pele principles.’

‘… without checking on the consequences the patients were informed of the Batho Pele principles. Now the patients want to hear what they want to hear.’

Planning affords an organisation a formal structure which will lead to better coordination of activities and, in the context of this article, better service delivery. In a study of cross-sectional surveys of nurses and patients to explore patient safety, satisfaction and quality hospital care in 12 countries of Europe and the United States of America, conducted and published by Aiken *et al*. (2012), results show that an organised healthcare worker environment is closely assosciated with quality care and patient and nurse satisfaction. For this reason the participants felt that the Batho Pele principles needed to be revisited.

#### Sub-theme 1.3: Introduction of Batho Pele principles affected by shortage of human and material resources

The participants indicated that besides the lack of planning by the relevant authorities, there were also human and material resource constraints which made it difficult for them to achieve the goals of the Batho Pele principles. Inadequate resources can have a negative impact on provision of care, leading to delays and deaths of patients. When there are no resources nurses are unable to render quality care and give of their best (Yoder–Wise 2007). The participants reported that they often have to rely on crisis management. In the patients’ eyes this is often perceived as non-caring inefficiency. A lack of resources, such as staff shortages, means patients might not always get their treatment on time or are sent home to return to collect outstanding medication later.

Aiken *et al*. (2012) argue that provision of resources (staffing and materials) improves patient outcomes as there is less occurence of staff burnout and patient complaints. Furthermore, Hellriegel *et al*. 2002) state that managers need to identify and commit the organisation's resources to achieving its goals. In view of Hellriegel's statement, Benjamin (2008) recommends that managers develop project management Gantt charts to help them focus on tracking tasks and allocating resources, thus identifying shortcomings and providing the necessary help. The following are some of the statements made by participants in relation to inadequacy of material resources:

‘Most of the time we nurses we improvise or go round borrowing from other departments or hospitals because there are no resources.’

‘Nurses would like to work and produce good results. When there are no resources I feel disgruntled, fed up. I feel like taking my bag and resign[*ing*] and find[*ing*] work where there are resources and I can give optimal care.’

Caring promotes patient satisfaction. For patients, caring denotes positive connectedness, being instructed and taught, and having a nurse spending time with them and being patient with them (Palese *et al*. 2011:341). In the current study participants perceived themselves as not fulfilling this expectation due to limited materials, resulting in conflict between themselves and their patients.

### Theme 2: Owing to introduction of the Batho Pele principles, nurse-managers are letting the professional nurses down

Participants felt that they were not receiving enough support from their managers, expressed in statements such as that below:

‘To report a patient or even a staff member who threatens you, you will be wasting time. Nothing happens.’

Dessler (2004) argues that a manager is a person who plans, organises, leads and controls the work of others so that the organisation can achieve its goals. If the manager fails to adhere to these attributes the department will be chaotic and nursing care will be compromised (Dessler 2004).

In their recent study Aiken *et al*. (2012) found that managerial support of nursing care, nurse participation in decision-making and organisational priorities on care quality were directly associated with nurse-workforce outcomes and nurses who were intent on leaving or remaining in their nursing jobs. This is in keeping with the perceptions of the participants in this study. The finding of that study can be applied in this current study, as the participants perceived the nurse-managers as not supportive of their actions, and at times were embarrassed in front of the patients as the nurse-managers took sides with the patients.

#### Sub-theme 2.2: Batho Pele principles cause the nurse managers to lack discipline in their leadership

In the participants’ opinion challenges to introduction of the Batho Pele principles were further aggravated by inconsistencies in the application of disciplinary measures by nurse managers – yet there were appropriate policies and guidelines in place. The participants reported that such policies and guidelines were not adhered to:

‘The disciplinary policies are there but they are not implemented, especially if that person is a shop steward nothing is done. They do as they please. They are rewarded for their behaviour, like they will be promoted or sent for courses.’

‘There is no discipline in our hospitals, everyone does what he/she likes, and even if you report that person nothing happens.’

Owing to this lack of disciplinary consistency, everyone could do as they pleased. This finding is similar to what was found in a similar study conducted in Ekurhuleni District of Gauteng Province, SA. That study revealed that nurse managers are to be blamed at times for non-supervision of staff performance, lack of role-modelling, and ineffective planning and control of their nursing units, all of which negatively affects implementation of the Batho Pele principles (Khoza, Du Toit & Roos 2010). However, ill-discipline was not only related to inadequately upheld disciplinary measures but also to matters concerning professionalism.

### Theme 3: Doctors are not committed to implementation of the Batho Pele principles

Doctors have the same responsibility as the nurses with regards to upholding the Batho Pele principles; however participants felt that sometimes their actions belied this.

**Sub-theme 3.1:** Doctors perceived as being impolite to patients and nurses.

Most of the participants felt that some doctors were extremely impolite and sarcastic. Participants had experienced doctors shouting at them in front of patients when they queried their behaviour or prescription. Participants felt that some doctors ignore the fact that a nurse is part of the health team:

‘Batho Pele is for all categories, not only for nurses.’

‘Doctors must get lectures on the upholding of the Batho Pele principles.’

Searle (2006:172) contends that nurses act in the interests of the patient and have a joint responsibility with the doctor for ensuring that the patient receives the correct prescribed diagnostic and therapeutic treatment. It is therefore important for doctors to familiarise themselves with the contents of this policy.

### Reaching the research goals

The research conducted was guided by two objectives, which were accomplished at the end of the study. Participants shared their perceptions relating to introduction of the Batho Pele principles. Through the use of exploratory questions, participants were able to provide in-depth information related to their perceptions, that could be both described and analysed. Findings revealed that professional nurses are overwhelmed by challenges resulting from the introduction of these principles. According to the participants, the objective of introduction of these principles was good, but implementation of the introduction of the Batho Pele principles lacked proper planning.

Data were analysed and the findings were used to develop guidelines that will be recommended to hospital managers as a means to facilitate realisation of the objectives of the Batho Pele principles within State hospitals.

## Limitations of the study

The concept of the Batho Pele principles is fairly new in the South African healthcare system and, like any other change introduced in a system, it has been confronted with some challenges. The researcher experienced it as being limiting to having to only interview professional nurses who work in outpatients’ departments, excluding professional nurses from other departments and wards of the hospitals in the study.

## Recommendations

A second objective of this study was to recommend measures that could be used for facilitation of all the parties involved in the realisation of the objectives of the Batho Pele principles. Based on the findings of the study, the guidelines outlined below are recommended to facilitate effective implementation of the Batho Pele principles.

### Guideline 1: Planning of health services

The researcher believes that the staff would be eager supporters of the Batho Pele principles if:

- they could be assured of their access to the necessary human and material resources, as well as to an adequate and reliable public and emergency services transport system for the purpose of speedy referrals;
- the hospitals have fully functional Accident and Emergency Departments to allow for direct referrals;
- all the hospitals are provided with the necessary security measures to safeguard the environment for both patients and staff; and
- all stakeholders are provided with educational programmes and other formal means to inform them about the standards of care they will receive.

### Guideline 2: Effective leadership and management

The hospital and nurse managers should and are to:

- be sensitive to employees’ problems and show genuine concern;
- create an environment in which employees are able to render optimal care, a non-threatening environment characterised by safety and security, where discipline is the order of the day;
- ensure orientation of new personnel to the policies of the institution, basing all punitive discipline on accepted legal prescriptions and remaining uninfluenced by other staff members or unions in consistency in implementing discipline; and
- be exemplary and keep to issues related to punctuality and work attendance, in keeping with the necessary institutional policies.

### Guideline 3: Availability of human and material resources

There are never enough resources for the work that needs to be done. In order to overcome this gap managers need to be:

- well-versed in financial management and budgetary control; and
- able to prioritise and be cost-effective without compromising quality care.

To achieve the clinical-related recommendations the researchers provide a further guide, based on the principles informed by the article ‘Healthcare in a land called People Power: nothing about me without me’ (Delbanco *et al*. 2001:144). A summary of those principles is listed below:

- Collaborative partnerships with relevant stakeholders.
- Approach the groups identified and inform them of the intentions of the collaboration.
- The groups should be represented by non-governmental organisations, lay persons, patients, doctors, multi-disciplinary teams, nurse managers and, where possible, legal staff.
- Allow involvement at decision-making level, as this allows for stakeholder commitment and ownership.
- Share information with interested groups.
- Identified groups are to be informed of the changes and interpretation of the changes so as to allow them to take that information to the masses.
- Information should also relate to the objectives of (in this instance) the Batho Pele principles.
- Collaborations with community-based leaders.
- Lay, retired and other well-respected, experienced people in the community could be approached for assistance in dealing with misinformation and incorrect perceptions of the introduction of the Batho Pele principles.
- In-service education.
- Nurse managers or persons in these positions should be continuously guided in their roles.
- Nurse managers need also to be guided as to how to motivate surbordinates, for example by using incentives.
- Nurse managers should be trained on how to be adequate role models.

## Conclusion

Batho Pele is a well-founded strategy towards a quality healthcare service, but is posing a challenge in the South African healthcare context. A revised strategy is needed to encourage ownership and uptake of these principles by key stakeholders, namely professional nurses, managers and the community.

The Batho Pele principles are intended for empowerement of the community through developing responsible attitudes towards health care and usage of healthcare services. From the findings of this study it is evident that this objective has not yet been achieved, owing to the diverse factors discussed in this article. The implication is that funds are still not being adequately channelled or used correctly. It is possible that because of the funds not being used correctly, some patients are still not getting the care they need and, as a result, are prone to complications that could lead to either chronic conditions or longer stays in hospital. Both of the latter ultimately lead to the hospital losing more funds.

Notwithstanding the perceptions of participants with regard to introduction of the Batho Pele principles, the researcher saw it necessary to recommend measures that could facilitate enhancement of implementation of these principles. Three guidelines were developed from the study, in the hope of ultimately developing permanent policies to guide implementation of the Batho Pele principles.
